# Functional Analysis of Prognostic Gene Expression Network Genes in Metastatic Breast Cancer Models

**DOI:** 10.1371/journal.pone.0111813

**Published:** 2014-11-04

**Authors:** Thomas R. Geiger, Ngoc-Han Ha, Farhoud Faraji, Helen T. Michael, Loren Rodriguez, Renard C. Walker, Jeffery E. Green, R. Mark Simpson, Kent W. Hunter

**Affiliations:** Laboratory of Cancer Biology and Genetics, Center for Cancer Research, National Cancer Institute, National Institutes of Health, Bethesda, Maryland, United States of America; University of Alabama at Birmingham, United States of America

## Abstract

Identification of conserved co-expression networks is a useful tool for clustering groups of genes enriched for common molecular or cellular functions [1]. The relative importance of genes within networks can frequently be inferred by the degree of connectivity, with those displaying high connectivity being significantly more likely to be associated with specific molecular functions [2]. Previously we utilized cross-species network analysis to identify two network modules that were significantly associated with distant metastasis free survival in breast cancer. Here, we validate one of the highly connected genes as a metastasis associated gene. *Tpx2*, the most highly connected gene within a proliferation network specifically prognostic for estrogen receptor positive (ER+) breast cancers, enhances metastatic disease, but in a tumor autonomous, proliferation-independent manner. Histologic analysis suggests instead that variation of TPX2 levels within disseminated tumor cells may influence the transition between dormant to actively proliferating cells in the secondary site. These results support the co-expression network approach for identification of new metastasis-associated genes to provide new information regarding the etiology of breast cancer progression and metastatic disease.

## Introduction

Advances in sequencing and computational technologies have enabled biologists to examine the complex inter-relationships between genes in an unprecedented scale. High throughput technologies such as gene chip or RNA-sequence analysis permit investigators to determine how perturbations affect the transcriptional program of entire genomes, rather than select pathways. If sufficient numbers of samples are examined, correlations between genes can be determined and groups of highly correlated genes can be visualized as gene network “modules” [2]. The modules are frequently enriched for genes mediating particular molecular and/or cellular functions [1], which can be used to implicate specific biological processes in phenotypes of interest. In addition, network analysis can implicate novel or poorly annotated genes with particular biological functions or phenotypes based on statistically significant transcriptional correlations.

Previously we have utilized gene network analysis to investigate the transcriptional programs associated with breast cancer metastasis [3]. Metastasis, the colonization and growth of secondary tumors at sites distant from the primary lesion, remains a significant problem for the management of human neoplastic disease. It is estimated that 90% of cancer mortality associated with solid tumors in humans is the result of metastatic disease rather than the primary tumor [4]. Better understanding of the etiology of tumor progression and metastasis is therefore important in the development of improved metastasis prevention strategies and anti-metastasis therapies.

**Figure 1:**
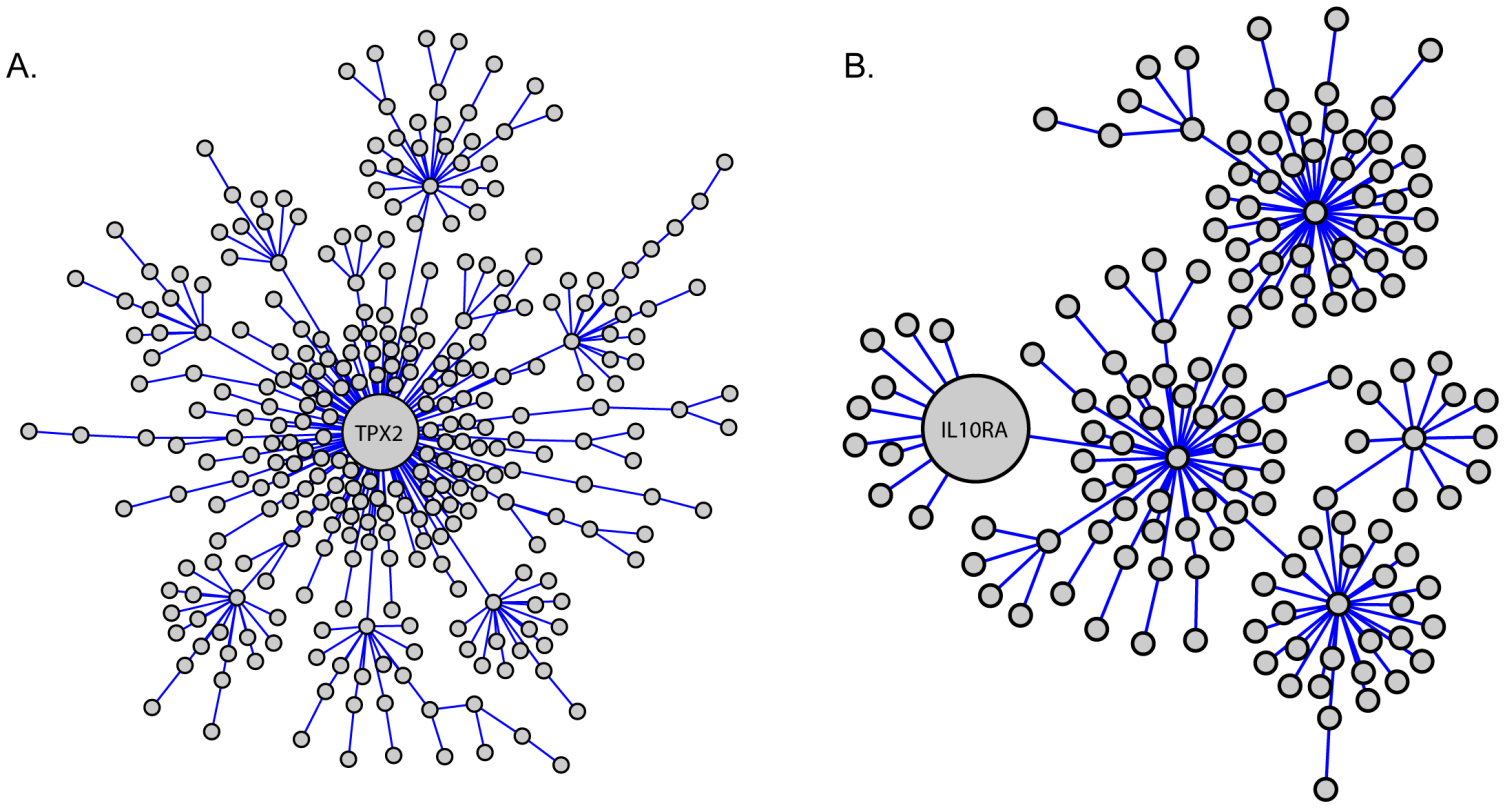
Network modules from the cross species analysis. A) Proliferation associated network centered around the *Tpx2* gene. B) Immune cell network containing the *Il10ra* gene. Both Figures are adapted from Hu et al. [3].

In our previous studies we utilized a cross species network analysis to identify gene co-expression network modules that were conserved between both mouse mammary tumors and human breast cancers [3]. These gene networks were subsequently screened on human breast cancer expression data to identify those modules that were able to predict distant metastasis free survival (DMFS) in patients. Two network modules were identified that reproducibly predicted DMFS. A module that was enriched for cellular proliferation genes, centered on the microtubule-associated gene *TPX2* was found to be prognostic for estrogen receptor-positive (ER+) breast cancer (Figure 1). The second network module was enriched for immunologically related genes and was prognostic specifically for the estrogen receptor-negative (ER−) class of human breast cancer (Figure 1B). Proliferation [5]–[7] and immunologic gene signatures [8], [9] have been previously associated with progression in metastatic breast cancer. However, membership of a gene within these transcriptional network modules does not necessarily imply a causative role in either the establishment of the co-expression network module or the phenotype of interest. The network modules might also be the result of subtle changes in upstream factors, for example transcription factor levels or post-transcriptional modifications, whose downstream effects are amplified to generate the network module, but do not encompass the primary causative factor.

It is therefore necessary to directly test individual genes present in co-transcription modules for any potential causative role in the generation of the network modules or biological phenotypes. To that end, in this study we investigate the etiological role of *Tpx2* in the establishment of the conserved prognostic expression network module and metastatic progression. We demonstrate that *Tpx2*, the most highly connected gene within the conserved proliferation gene network module, does not play a role in the establishment of the co-expression network. *Tpx2* is however, causally associated with metastatic progression in a model of human ER+ breast cancer. Proliferation-related genes are frequently associated with prognostic genes signatures [10] which suggest that proliferative capacity might be causally associated with metastatic disease. However, despite being the central node of the proliferation associated network module, the role of TPX2 in metastatic breast cancer can be independent of a role in tumor cell proliferation rates. This suggests that mechanisms mediating metastasis may be more sensitive to cell cycle gene-related dosage than cell proliferation rates and implies potential additional cellular functions for at least some of these genes.

## Materials and Methods

### Generation of Tpx2 knockdown cell lines

Lentiviral short hairpin RNA-interference vectors targeting Tpx2 were part of the TRC collection [11] and purchased from Thermo Scientific: shTpx2#1 corresponds to TRCN0000120812 (target sequence: CCAGACTTCTTGTAGTTATTT), shTpx2#2 corresponds to TRCN0000120814 (target sequence: GCTCAACCTGTGCCACATTAT), shScrmbl is a non-targeting scrambled control vector obtained from Addgene (plasmid #1864, hairpin sequence CCTAAGGTTAAGTCGCCCTCGCTCGAGCGAGGGCGACTTAACCTTAGG) [12]. 6DT1 mouse mammary carcinoma cells derived from an MMTV-c-Myc transgenic mouse model [13] and HEK 293 cells (ATCC) were grown in DMEM (Gibco) +9% Fetal Bovine Serum (Gemini Bio-Products) +2 mM Glutamine (Gibco) and penicillin/streptomycin (Gemini Bio-Products) at 37°C in a humidified atmosphere with 5% CO_2_. Lentiviral particles were generated by transfecting logarithmically growing HEK293 cells with 0.75 µg psPAX2 (plasmid #12260), 0.25 µg pMD2.G (plasmid # 12259, both from Addgene [14]) and 1 µg of the respective pLKO.1 plasmids, using Xtremegene9 transfection reagent (Roche) according to the manufacturer’s instructions. 48 h after transfection virus-containing supernatant was passed through a 0.45 µl filter (Millipore), transferred to logarithmically growing 6DT1 cells and incubated for 16 h in the presence of 5 µg/ml polybrene (Sigma). Stable, polyclonal cell pools were obtained by selection with 3 µg/µl puromycin (Sigma) for 5 days.

### Quantitative real time (qRT−) PCR

Total RNA was isolated from cells with the RNeasy kit (Qiagen) and cDNA synthesized from 2 µg (4 µg for E-cadherin/*Cdh1* analysis) total RNA with iScript cDNA Synthesis Kit (BioRad). qRT-PCR was performed on a 7900 HT Fast Real Time PCR System (Applied Biosystems) using SYBR Green (USB Affymetrix). Peptidylprolyl isomerase B (*Ppib*) was used for normalization of expression levels and statistical significance determined by Student’s T-test. Primers used were: *Bub1*-F: CCACTTGGAGAATGGGAAAGC, *Bub1*-R: GGTCACTGTTGTACTCAGCAAA, *Bub1b*-F: GAGGCGAGTGAAGCCATGT, *Bub1b*-R: TCCAGAGTAAAAGCGGATTTCAG, *Ccnb2*-F: CAGTGACTACGTGAAGGACATC, *Ccnb2*-R: TGGCACGCATACGTCCATTTA, *Cdc20*-F: TTCGTGTTCGAGAGCGATTTG, *Cdc20*-R: ACCTTGGAACTAGATTTGCCAG, *Cdh1*-F: TCCTGCCATCCTCGGAATC.

*Cdh1*–R: CTGTGCAGCTGGCTCAAATC *Cenpa*-F: CTCCAGTGTAGGCTCTCAGAC, *Cenpa*-R: CTGAAAGGCTTCTTCCTGAACA, *Cep55*-F: CCTAGTAGCTCCAAGTCAGACA, *Cep55*-R: ACCTTAGGTGGTCTTTGAGTCTC, *Kif2c*-F: ATGGAGTCGCTTCACGCAC, *Kif2c*-R: CCACCGAAACACAGGATTTCTC, *Ppib*-F:GGAGATGGCACAGGAGGAAAGAG, *Ppib*-R:TGTGAGCCATTGGTGTCTTTGC, *Tpx2*-F: GATGAGCGAATCAAGCAACATC, *Tpx2*-R: GCTTAATGATAGTGCATCCTCTGGTT, *Ube2c*-F: CTCCGCCTTCCCTGAGTCA, *Ube2c*-R: GGTGCGTTGTAAGGGTAGCC.

### Animal experiments

8 weeks old female FVB/NJ mice (Jackson Laboratories, Bar Harbor) were injected with 1×10^5^ 6DT1 cells into the fourth mammary fat pad. Animals were euthanized and dissected 27 days after injection. Tumors were weighted and lungs inspected by eye for the presence of metastatic nodules. Statistical significance was calculated with a Kruskal-Wallis test followed by Conover Inman test. All animal experiments were performed in compliance with the National Cancer Institute's Animal Care and Use Committee guidelines.

### Kaplan Meier survival curves

GOBO (Gene expression-based Outcome for Breast cancer Online) is an online tool (http://co.bmc.lu.se/gobo/) with data from 1225 ER-positive human mammary tumors [15]. Inputs for the analysis were: Screen upload of gene set: TPX2, Tumor selection: ER+, Select number of groups (quantiles): 3, Select censoring (years): 10, Select end-point: Distant Metastasis-free Survival (DMFS).

### Immunohistochemistry and semiquantitative in-vivo proliferation analysis

Tissues were fixed in 10% formalin, embedded in paraffin, sectioned and stained for Ki67 at the Frederick National Laboratory for Cancer Research, Laboratory Animal Sciences Program, Pathology/Histotechnology Laboratory with citrate heat-induced epitope antigen retrieval and 1∶100 anti Ki61 antibody (ab16667, Abcam) and ABC DAB chromogen. Slides of stained sections were scanned with an Aperio XT digital scanner. Manual segmentation was used to select total tumor areas within the implanted tumor samples and lung sections for each specimen. Ki-67 immuno-labeling was quantified using the Aperio Image Analysis Toolbox Software nuclear algorithm. Positive nuclei were identified by user-defined parameters for size, shape, and chromogen label intensity. Acquisition of a minimum of 10,000 tumor cells was set for the analysis, except for one lung sample of shTpx2#1 that displayed very small metastases and failed to meet the minimal number of tumor cells. All samples were visually inspected to ensure errors were within acceptable limits.

### In-vitro cell proliferation assays

For long-term proliferation assays, cells were seeded in triplicate at equal densities and passaged every 3–4 days. At each passage, cells were counted, total number of cells calculated, and subsequently re-plated at equal densities. Short-term proliferation assay was carried out on an Incucyte live content imaging system (Essen Bioscience) by seeding 2000 cells in quadruplicates on 48-well cell culture plates and imaging every 2 h for up to 160 h. For BrdU incorporation assay 10^6^ cells were seeded on 6 cm cell culture dishes and 24 h later 10 µM BrdU (5-bromodeoxyuridine, Sigma) added for 25 min followed by trypsinization and fixation in 70% ethanol for at least 30 min at 4°C. Cells were then treated with 0.5 mg/ml RNaseA (Invitrogen) for 30 min at 37°C, treated with 5M HCl +0.5% Triton X-100 for 20 min at room temperature, and neutralized with 1M TRIS pH 7.5. After washing with PBS +0.5% Tween-20 cells were incubated with anti BrdU-FITC (eBioscience, clone BU20A) for 30 min at room temperature, washed twice with PBS +0.5% Tween-20, and finally resuspended in PBS +20 µg/ml propidium iodide(PI, Thermo Scientific) for staining of DNA. Samples were analyzed by fluorescence-activated cell sorting (FACS) on a FACSCALIBUR cytometer (BD Bioscience) and data processed with FlowJo software.

### Western blot analysis

67NR and 4T1 cells were a gift from L. Wakefield (NCI, Bethesda [16]). Protein was extracted by cell lysis on ice for 30 minutes in Golden Lysis buffer (10 mM Tris pH 8.0+400 mM NaCl +1% Triton X-100+10% Glycerol+Complete protease inhibitor cocktail (Roche)+phosphatase inhibitor (Sigma)). 30 µg (or as indicated) total protein extract in NuPage LDS Sample Buffer (Invitrogen) and 0.25% beta-mercaptoethanol were resolved on 3%–8% Tris-Acetate SDS-PAGE (NuPage, Invitrogen), transferred onto PVDF membrane (Millipore), blocked in 5% skim milk and incubated overnight with the following primary antibodies at 4°C: mouse anti E-cadherin (BD) 1∶5000, mouse anti beta-catenin (BD) 1∶1000, mouse anti vimentin (Sigma) 1∶5000, and mouse anti beta-actin (Abcam 1∶10000. Membranes were then incubated with horse-radish peroxidase linked anti-mouse (GE Healthcare) and immunoblots visualized using Amersham ECL Prime Western Blotting Detection System and Amersham Hyperfilm ECL (GE Healthcare).

### Anoikis assay

Cells were plated in ultra-low adhesion plates (Costar) in triplicate or quadruplicate in complete media. Plates were then incubated for 7 days before cells were harvested and counted using a Nexcelom Cellometer Auto T4. Cell numbers for each sample were averaged from four independent experiments and normalized to the shCtrl control. P values were calculated using GraphPad Prizm.

### Wound healing assay

Cells were plated in Essen ImageLock 24-well plates and grown until confluent. Once the cells were confluent the media was aspirated, and then incubated 3–4 hours with complete media containing 10 ug/ml Mitomycin C (Sigma). Scratch wounds were then made using the Incucyte Scratch instrument and displaced cells and debris removed by washing with PBS^-^. The plates were re-fed with complete media, placed in the Incucyte Kinetic Live Cell Imaging System (Essen BioScience), and cell motility imaged for 72 hours.

### Immunofluorescence

Cells were grown on sterile glass coverslips, fixed in 4% paraformaldehyde for 20 min, and permeabilized and blocked with PBS +0.05% Triton-X 100 (PBST) supplemented with 1% BSA for 30 min. Primary antibodies were: mouse anti E-cadherin (BD) 1∶500 in PBST +1% BSA for 1 h. Secondary antibody was Alexa Fluor 594 goat-anti mouse (Invitrogen), actin co-staining was done with phalloidin-Alexa Fluor 488 (Invitrogen) 1∶200, both diluted in PBST +1% BSA and incubated for 30 min. PBST was also used for washing slides in between steps; all procedures were carried out at room temperature. Slides were mounted with Vectashield (Vector) and analyzed on a Zeiss LSM 710 NLO confocal microscope with Zeiss ZEN software.

### Metastasis size quantitation

Butterfly section of mouse lungs stained with H&E and scanned using an Aperio slide scanner. Macro-metastases were defined by those lesions that could be scored by eye. Micro-metastases were scored by analysis using the ImageJ software package.

### Ethics Statement

All animal experiments were performed in accordance with the guidelines of the National Cancer Institute Animal Care and Use Committee, under the approved Animal Study Protocols LCBG-002 or LCBG-004. All animal surgeries were performed with the approved anesthetics and analgesics to minimize animal discomfort.

## Results

### Tpx2 knockdown impairs metastasis without affecting tumor growth in 6DT1 cells

**Figure 2:**
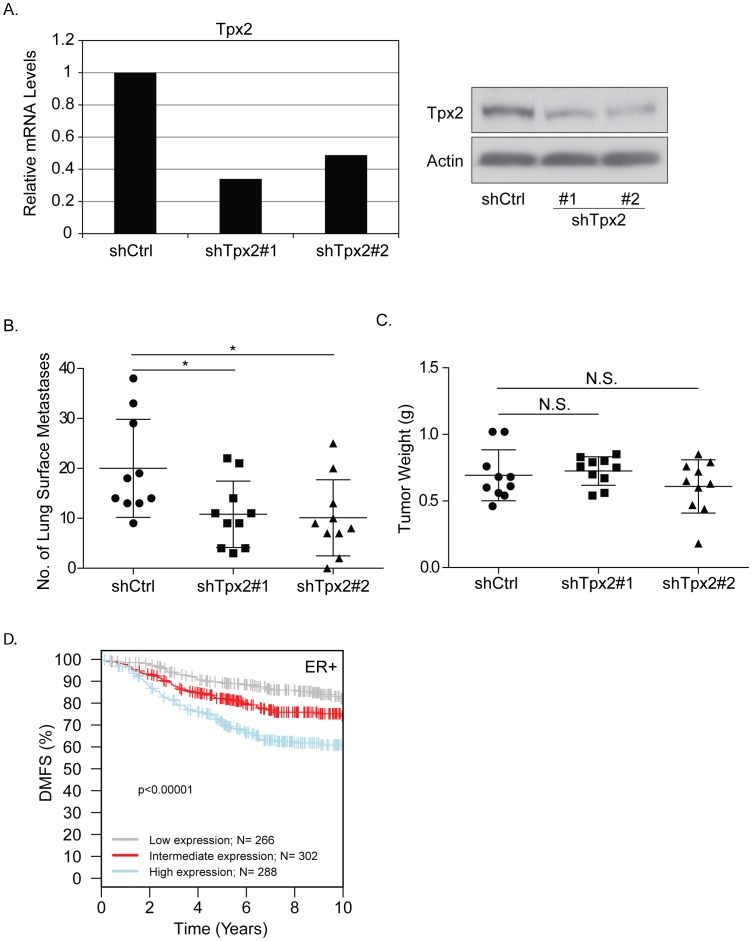
Tpx2 knockdown significantly affects metastasis but not tumor proliferation. A) Relative expression levels of *Tpx2* in 6DT1 cells as measured by qRT-PCR and western blotting. B) 1×10^5^ 6DT1-shTpx2 or -shCtrl cells were orthotopically injected into the mammary fat pad of female FVB mice. Mice were euthanized and lungs dissected and inspected for metastatic nodules on the surface of the lungs 27 days after injection. Asterisks indicate a p-value <0.05. C) Weight of primary tumors dissected from the mammary fat pad of mice described in A. No significant differences (N.S.) were observed. D) The Gene expression-based Outcome for Breast cancer Online (GOBO) database was queried for Tpx2 and distant metastasis-free survival (DMFS) plotted as Kaplan-Meier curves for patients with ER-positive tumors expressing high (blue), intermediate (red), or low (gray) levels of *TPX2*.

**Figure 3:**
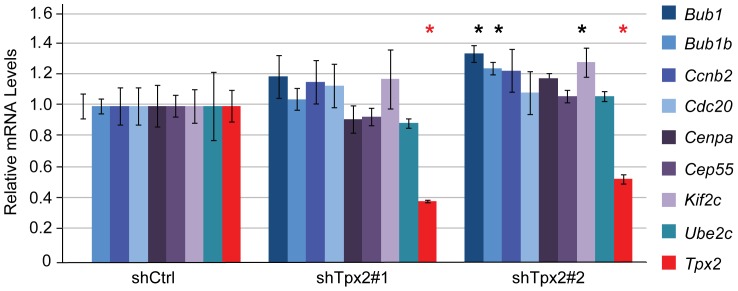
Partial Tpx2 ablation in 6DT1 cells does not change expression levels of Tpx2 network hub genes. 6DT1 mouse mammary carcinoma cells were lentivirally transduced with pLKO-Scrambled (shCtrl), pLKO-Tpx2#1 (shTpx2#1), or pLKO-Tpx2#2 (shTpx2#2) and stable, polyclonal pools generated. qRT PCR was carried out to measure mRNA levels of *Tpx2* and eight genes previously identified as ‘hubs’ of the Tpx2 gene expression network [3]. Gene expression levels are displayed relative to levels in shCtrl control cells, error bars represent standard deviations, asterisks indicate p-values <0.05. Only *Tpx2* was significantly down regulated in both shTpx2 cell lines (red asterisks).

Previously *TPX2* was identified as the most highly connected node in a gene network that predicted distant metastasis free survival (DMFS) in ER+ breast cancers (Figure 1a; [3]). To determine whether *TPX2* contributed to the DMFS discriminatory capacity of this network shRNA knockdown of *Tpx2* was performed in a highly metastatic mouse mammary cell line, 6DT1 [13] originally derived from an MMTV-myc transgenic animal, which gene expression analysis suggests most closely resembles human luminal breast cancer [17] which form ER+ tumors after orthotopic implantation [18]. *Tpx2* was partially depleted in 6DT1 cells by lentiviral transduction with two independent, non-overlapping short-hairpin RNA (shRNA) interference constructs and stable, polyclonal pools were used in all subsequent experiments. Figure 2A shows *Tpx2* expression levels at 50%−70% of that of control cells at mRNA as well as protein level. Since *Tpx2* expression levels are usually tightly controlled within cells the partial suppression more closely mimics the physiological and pathological situation resulting from expression variation due to polymorphism than a complete knockout. Orthotopic implantation of the 6DT1-shTpx2 cells into the mammary fat pad of female FVB mice resulted in significant reduction of pulmonary metastasis compared with 6DT1 cells expressing a non-targeting short hairpin control (Figure 2B). This result was consistent with human patient data queried using the Gene expression-based Outcome for Breast cancer Online (GOBO) database [15]. Patients whose tumors had lower *TPX2* expression levels showed statistically significantly increased distant metastasis-free survival than those with higher *TPX2* expression levels (Figure 2D). Strikingly, in the orthotopic breast cancer transplantation model there was no difference in the size of the primary tumors between shTpx2 and control shRNA (Figure 2C), indicating that tumor cell proliferation was not impaired. This was unexpected since Tpx2 is known to be functionally involved in mitotic spindle checkpoint regulation. Furthermore gene signatures that are prognostic for ER+ breast cancers are thought to primarily measure tumor aggression as a function of proliferative capacity. These results therefore raise the possibility that despite its association with the cell cycle Tpx2 can regulate metastasis independently of its known function in proliferation.

### Partial Tpx2 ablation in 6DT1 cells does not change expression levels of Tpx2 network hub genes

The central position of *TPX2* in the gene network suggests the possibility that variations in TPX2 levels might directly influence the transcriptional output of the other network members. However, since the *TPX2* network consists primarily of cellular proliferation genes, the lack of difference between primary tumor size in the transplant experiments would suggest that *TPX2* is not driving the transcriptional network but instead is merely associated with the expression of the network genes. To test this we therefore compared the expression levels of eight previously described ‘hub’ genes (*Bub1, Bub1b, Ccnb2, Cdc20, Cenpa, Cep55, Kif2c, Ube2c)* that are most closely correlated with the rest of the network, between 6DT1 shTpx2 and control cells. None of the hub genes were significantly and consistently changed upon knockdown of Tpx2 (Figure 3), suggesting that Tpx2 is not a master regulator of the network.

### Partial Tpx2 suppression does not impair 6DT1 cell proliferation in vitro

**Figure 4:**
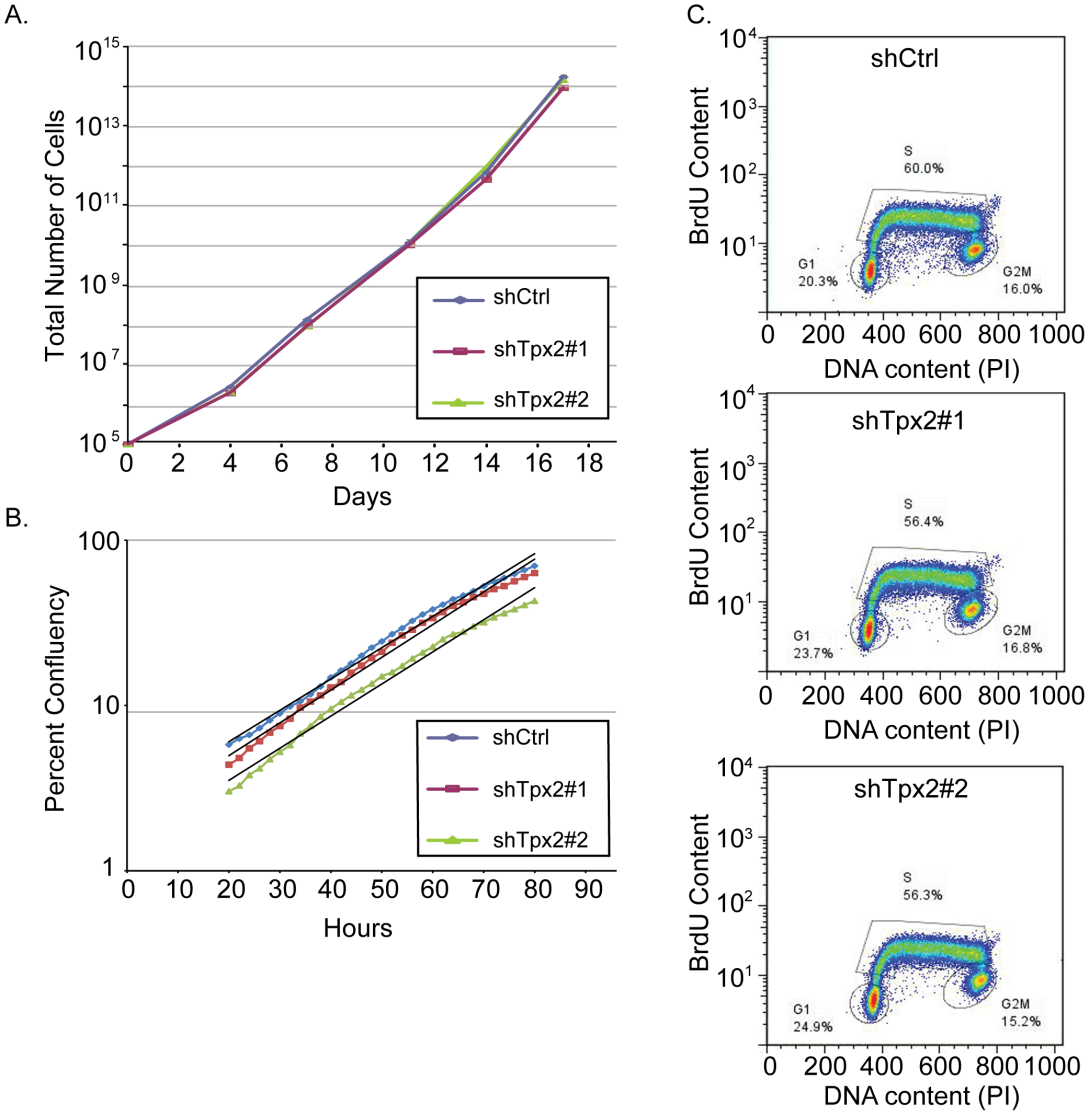
Knockdown of Tpx2 does not impair 6DT1 cell proliferation in vitro. A) 6DT1-shCtrl, 6DT1-shTpx2#1 and 6DT1-shTpx2#2 cells were seeded in triplicate at equal densities and passaged every 3–4 days for 17 days. Cumulative cell numbers were determined at each passage. B) 2000 cells were seeded in quadruplicates into 48-well cell culture plates and imaged for >80h. Average cell density during logarithmic growth from 20 h–80 h after seeding is displayed and exponential trend lines are shown in black. C) Exponentially growing cells were pulsed with 10 µM BrdU for 25 min, stained for DNA and BrdU content and analyzed by FACS. Percentage of cells in G1, S, and G2/M phase are indicated.

Next we investigated whether knockdown of *Tpx2* in 6DT1 cells affects proliferation in vitro. *In vitro* growth curve analysis revealed no difference in the proliferation rate of 6DT1 shTpx2 and 6DT1 shRNA control cells (shCtrl) (Figure 4A), while *Tpx2* expression levels remained suppressed in 6DT1 shTpx2 cells throughout the 17-day time course of the experiment (data not shown). We also measured 6DT1 sh*Tpx2* and 6DT1 shRNA control cell proliferation in a short-term growth assay and, again, failed to measure any significant difference in cell proliferation rates (Figure 4B). Likewise, a BrdU incorporation assay did not show significant differences in the percentile distribution of cells in G1, S, or G2/M phase of the cell cycle (Figure 4C).

### Tpx2 reduction does not impair 6DT1 cell proliferation in vivo

**Figure 5:**
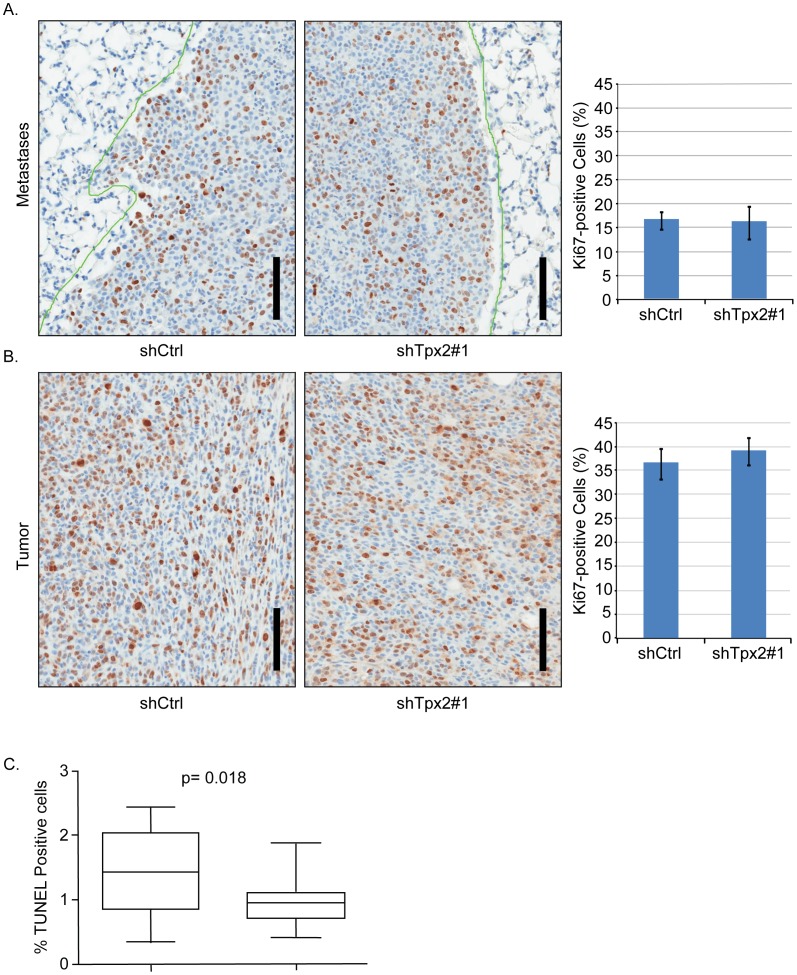
Knockdown of Tpx2 does not impair 6DT1 cell proliferation in vivo and does not affect anoikis. A) Lung sections from mice injected with 6DT1-shCtrl or 6DT1-shTpx2#1 harboring metastatic nodules and immuno-labeled with Ki-67 antibody. The bar diagram on the right represents quantification of percent tumor cell nuclei with immuno labeling (cycling fraction) relative to total numbers of tumor cells from five mice each. Error bars show standard deviations. B) Sections of primary tumors from mice injected with 6DT1-shRNA control or 6DT1-shTpx2#1 were stained and analyzed as in a). Scale bars correspond to 100 µm. C) shCtrl or *Tpx2* knockdown cells were plated into low adhesion plates, grown for 7 days, and viable cells quantified. The combined results of four independent experiments are represented. No significant difference in anoikis was observed by reduced *Tpx2* levels.

To more directly assess whether tumor cell proliferation was indeed not affected by knockdown of *Tpx2* in our model system we quantified proliferating cells in the metastatic lesions and primary tumors of 6DT1 shTpx2 and 6DT1 shCtrl-injected mice. To this end we stained histological sections for the proliferation marker Ki67 and quantified the percentage of Ki67-positive tumor cells. Consistent with our previous findings, no significant difference in tumor cell proliferation could be detected between 6DT1 shTpx2 and 6DT1 shCtrl in either metastases (Figure 5A) or primary tumors (Figure 5B). This finding further strengthened our hypothesis that the effect on metastasis by knockdown of Tpx2 was independent of its function in mitosis. We therefore concluded that, in 6DT1 cells, *Tpx2* knockdown impairs metastasis through a mechanism different from its known function in mitosis and cell proliferation.

### Suppression of metastasis by Tpx2 knockdown does not correlate with changes in tumor cell apoptosis

Work from other laboratories has indicated that the majority of disseminated tumor cells do not survive at the secondary site (ex. [19]). To determine whether the reduction in metastatic burden observed by *Tpx2* might be due to increased tumor cell apoptosis in the lung, TUNEL assays were performed. Semi-quantitative analysis of the primary tumors did not reveal any differences in TUNEL-positive cells between the control and knockdown cells (Data not shown). Due to diffuse necrosis in the control tumors fully quantitative analysis was not performed. To further examine the possible effect of apoptosis, TUNEL staining was also performed on lung metastases Interestingly, there was were significantly fewer TUNEL-positive cells in shTpx2 than control cells suggesting that the reduced number of metastases was not due to increased cell death in the lung (Figure 5C).

### Knockdown of Tpx2 does not reduce in vitro motility

**Figure 6:**
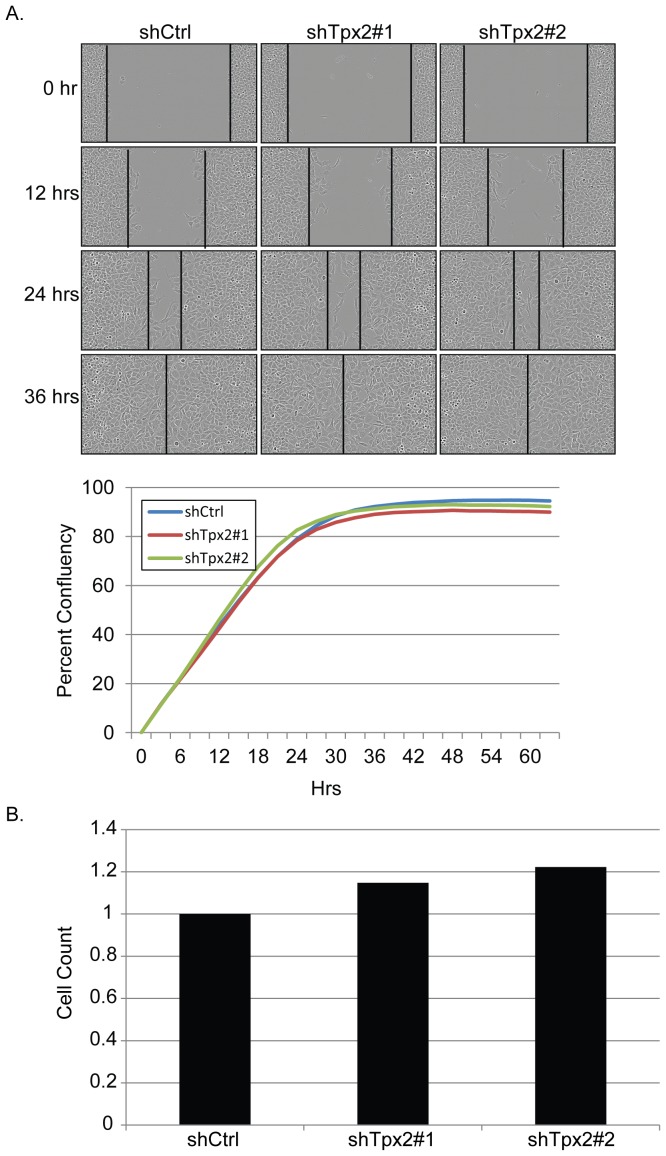
Reduction of Tpx2 does not increase wound-healing and apoptosis in 6DT1 cells. A) Representative photomicrographs of the shCtrl, *Tpx2* sh1 or *Tpx2* sh2 cells in the wound healing assay are shown at the top of the figure. A graphical representation of the assay, portrayed as percent confluence over time, is shown at the bottom of the figure. B) Percent of cells with positive labeling in the lung metastases for TPX2 control and knockdown lungs. For each sample, 4 quantification areas were set up in different metastatic nodules. Two knockdown samples did not have 4 metastatic nodules. In one sample, only one nodule is present. In the other sample, there were 3 nodules counted. Counting areas included either 2000–4000 cells or the entire nodule if it was less than 2000 cells. Areas with apparent off-target labeling due to necrosis were avoided, but single cell necrosis may be contributing to the increased labeling in the control metastases.

One possible explanation for the observed metastatic suppression by *Tpx2* knockdown might be a reduced ability of cells to escape the primary tumor. To address this possibility wound healing assays were performed. As seen in figure 6A no significant difference was observed between shCtrl and shTpx2 cells, suggesting migration defects were likely not the primary cause of *Tpx2*-knockdown suppression of metastatic disease.

### Tpx2 does not reduce metastasis through increased anoikis

The development of metastatic disease requires cells to survive anchorage-independence during transit through the vasculature or lymphatics from the primary to the secondary site. To test whether reduction of *Tpx2* might reduce metastatic burden by increasing anoikis, sh-Scrambled and sh-Tpx2 cells were plated on low adhesion plates and viable cell counts performed 7 days later. No significant difference was observed between the wild-type or knockdown cells, indicating that cell death in circulation was not likely to be a major contributor to the reduction of metastatic disease (Figure 6B).

### Tpx2 does not induce prototypical mesenchymal-epithelial transition (MET) in 6DT1 cells

**Figure 7:**
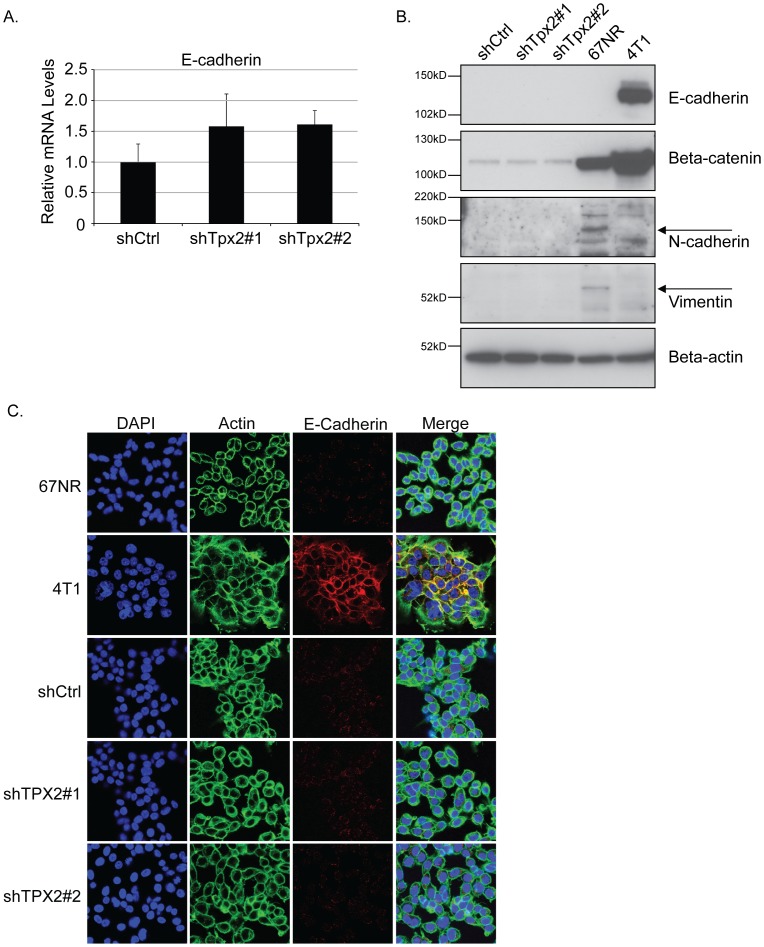
Knockdown of Tpx2 does not induce prototypical MET in 6DT1 cells. A) E-cadherin mRNA levels in 6DT1 shCtrl, 6DT1-shTpx2#1, and 6DT1-shTpx2#2 cells were measured by qRT-PCR and displayed relative to levels in shRNA control cells. Error bars represent standard deviations. B) Western blot analysis for E-cadherin, beta-catenin, N-cadherin, and vimentin of cell lines described in a). Beta-actin serves as loading control. 67NR and 4T1 cells serve as positive and negative controls for the different EMT markers. C) 6DT1-shCtrl, 6DT1-shTpx2#1 and 6DT1-shTpx2#2 cells (and 67NR and 4T1 cells as controls) were immunofluorescently stained for E-cadherin and actin cytoskeleton was labeled with phalloidin (green). DAPI was used for nuclear staining. Confocal images are shown at 63x magnification.

It has recently been shown that activated Aurora kinase can induce epithelial-mesenchymal transition (EMT) [20]. Since EMT is known to facilitate metastasis [21] and TPX2 has previously been shown to activate AURK [22], we speculated that Tpx2 depletion may impair metastasis through the reverse process, mesenchymal-epithelial transition (MET). To this end, we analyzed the expression levels of several epithelial and mesenchymal markers in 6DT1 shTpx2 and 6DT1 shRNA control cells. The mRNA levels of the epithelial marker E-cadherin were slightly elevated in 6DT1 shTpx2 cells (Figure 7A), however this did not translate into increased E-cadherin protein levels by either western blot analysis (Figure 7B) or immunofluorescence and confocal microscopy analysis (Figure 7C). Similarly, beta-catenin, another epithelial marker, was unchanged and the mesenchymal markers N-cadherin and vimentin were expressed below detection levels in both 6DT1 shTpx2 and 6DT1 shRNA control cells (Figure 7B). It thus appears that in the 6DT1 mammary carcinoma cells, Tpx2 functionally contributes to metastasis through an unknown mechanism, but independent of cell proliferation or EMT.

### Tpx2 knockdown may delay conversion from dormant to proliferative state in the secondary site

**Figure 8:**
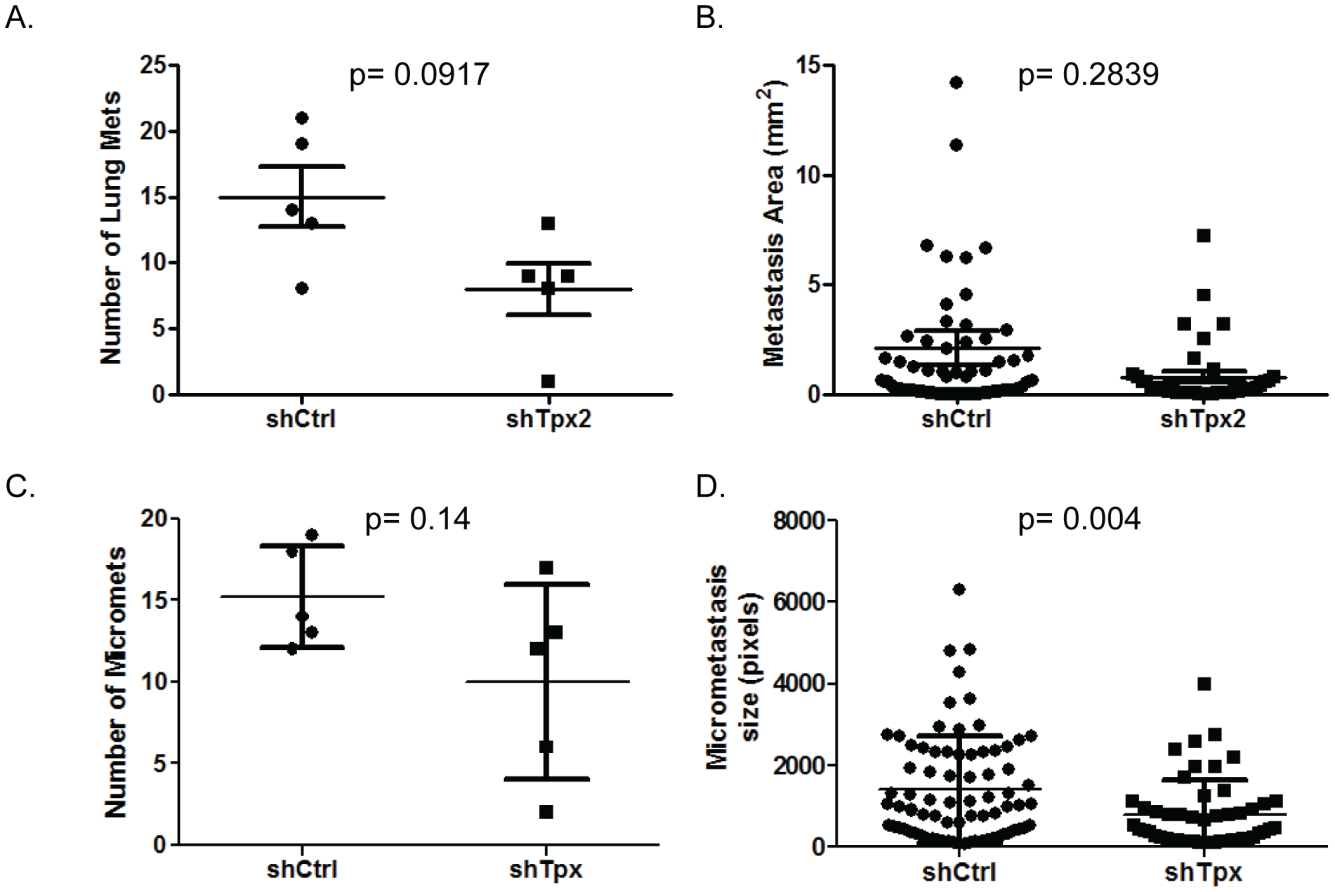
Quantitation of metastasis size from control or Tpx2 knockdown 6DT1 tumors. A) Comparison of the number of macroscopic metastases observed on H&E stained slides from control (shCtrl) versus knockdown (shTpx#1) cell lines. B) Surface area of macro-metastases (mm^2^) between control and knockdown samples. C) Number of micro-metastases observed for control and knockdown cells. D) Size of micro-metastases (pixels) observed for control versus knockdown samples.

Examination of the histology slides revealed that in addition to a reduction in the number of macro-metastases (Figure 8A), there was also a non-significant reduction in metastasis size (Figure 8B). This suggested that the reduction of metastases might be due to a reduction or delay in conversion of single dormant cells into proliferative lesions. If true, this would predict that the number and size of micro-metastases in addition to macro-metastases should be reduced. The slides were therefore scanned and evaluated for micro-metastatic lesions. As can be observed in figure 8C, the number of micro-metastases observed was trending downward in the shTpx2 samples, although not significant (p = 0.14) due to the high degree of variability in the knockdown samples. In contrast, the size of the micro-metastases in the knockdown samples was significantly reduced in the shTpx2 samples (Figure 8D: p = 0.0041).

## Discussion

We recently identified a gene expression network resembling a ‘proliferation signature’ and predicting distant metastasis free survival in ER+ breast cancer patients [3]. We now tested the central node of this network, Tpx2, and found that it is functionally involved in metastasis.

Unexpectedly, knockdown of *Tpx2* in the metastatic mammary carcinoma cell line 6DT1 did not change its proliferation rate in vivo or in vitro, as we had expected from the known role Tpx2 plays in mitosis. It therefore appears that Tpx2 has additional functions, independent of mitosis, but relevant for metastasis. The 6DT1 cells used in this study are derived from an MMTV-c-myc transgenic mammary tumor and are highly transformed, forming fast growing, highly metastatic tumors in mice [13]. It is likely that these cells have acquired additional mutations that may have rendered them independent of Tpx2 for mitotic progression, at least at the knockdown levels achieved in our experiments. This particular context could have unmasked the additional function of Tpx2 in metastasis, which has not been detected before in more benign tumor cells or in complete knockouts [23], [24] and suggests perhaps the mechanisms mediating TPX2-driven metastases are more sensitive to smaller changes in TPX2 levels than its role in proliferation. Of note, knockdown of *Tpx2* in Mvt1 mammary carcinoma cells impaired both primary tumor growth and pulmonary metastasis (data not shown), suggesting that in this context the known mitosis-related function of Tpx2 may be more critical.

Our work adds to the multiple indications that *Tpx2* is a tumor progression and metastasis-associated gene: *TPX2* is overexpressed in a number of cancers (for review see [25], [26]). In colon cancer Wei et al. found a correlation between increased *TPX2* levels and shorter metastasis-free survival [27]. Furthermore, interfering with *TPX2* in colon cancer cell lines decreased cell proliferation and also affected cell migration and invasion as well as expression of the metalloproteinase MMP-2 [27]. Statistical association between elevated *TPX2* levels and lymph node metastasis have been reported in esophageal squamous cell carcinoma [28] squamous cell lung cancer [28], medullary thyroid carcinoma [29], cervical cancer [30], and bladder carcinoma [31]. These observations raise the possibility that *TPX2* is a potential target for therapeutic intervention in multiple types of cancer, including ER+ breast cancer. While most of the above mentioned studies focused on the known function of *TPX2* in mitosis and proliferation, our work suggests that there may be additional functions of Tpx2 relevant to metastasis. Further studies will be required to determine whether *TPX2* is a useful therapeutic target or a prognostic or predictive marker that provides additional power to currently used clinical tools.

Interestingly, the mechanistic investigations suggest that changes in TPX2 levels most likely impacts the transition from single non-proliferative cells in the secondary site to proliferative lesions. Analysis of *in vitro* and *in vivo* proliferation of the tumor cells, migration, anoikis, EMT or apoptosis did not reveal any significant differences between control and knockdown 6DT1 cells. The only phenotypic difference observed was the average size of the macro- and micro-metastases. Since proliferation, as measured by Ki67 staining, was not significantly different at either the primary or secondary site, and apoptosis was the same at the primary site, and actually potentially lower at the secondary site for the knockdown compared to the controls, this suggests that the difference in metastasis size might be explained by a delay in conversion from a non-proliferative to proliferative state. This would result in a reduction in macroscopic metastasis during the experimental time course, as observed, and an increase in distant metastasis free survival in human patients, consistent with our and others previous observations. Exactly how variations in TPX2 levels result in this difference in proliferative state is currently unknown and will require additional investigations to begin to clarify the mechanistic basis of this phenomenon.

An important caveat of these studies however is the generalizability of the results. As mentioned above, the different phenotype of *Tpx2* knockdown in the 6DT1 and Mvt1 mammary cell lines does raise the possibility that the results observed here cannot be generalized across all breast cancers. Both 6DT1 and Mvt1 are derived from mouse genetically engineered models that are thought to be representative of human luminal breast cancers so if *Tpx2* is mechanistically associated in metastatic disease one might expect similar results across multiple cell lines. However, as mentioned above, the differences in metastatic and proliferation phenotype of *Tpx2* may depend on the relative levels of the protein, and potentially different thresholds of protein, in the different cell lines. Relatively subtle differences in TPX2 levels in the cell lines might therefore be responsible for the difference in phenotypes. Despite this uncertainty, we believe that the results are consistent with TPX2 playing a role in metastatic disease in at least some fraction of human cancers since multiple shRNAs in the 6DT1 cell line result in the same phenotype. Additionally, the data is consistent with reports from the human cancer literature. Thus while this data is consistent with a role of TPX2 in human metastatic progression, we believe it should be considered preliminary, and as indicated above will require additional validation in independent studies.

In summary, we validated the *Tpx2* gene previously identified through cross species gene expression network analysis as a regulator of breast cancer metastasis. We acquired further evidence that tumor autonomous mechanisms provide important determinants of metastatic potential in ER+ mammary carcinomas and showed that *Tpx2* can regulate metastasis independently of proliferation. These findings underscore the relevance of comparative gene expression network analyses across strains and species, which may help to identify novel therapeutic targets aimed at reducing tumor progression and metastasis in breast cancer.
